# Drug interactions among older adults followed up in a comprehensive medication management service at Primary Care

**DOI:** 10.31744/einstein_journal/2019AO4725

**Published:** 2019-08-19

**Authors:** Tayane Oliveira dos Santos, Mariana Martins Gonzaga do Nascimento, Yone Almeida Nascimento, Grazielli Cristina Batista de Oliveira, Ursula Carolina de Morais Martins, Danielle Fernandes da Silva, Djenane Ramalho de Oliveira

**Affiliations:** 1 Centro de Estudos em Atenção Farmacêutica, Faculdade de Farmácia, Universidade Federal de Minas Gerais, Belo Horizonte, MG, Brazil

**Keywords:** Drug interactions, Aged, Medication therapy management, Potentially inappropriate medication list, Pharmacists

## Abstract

**Objective:**

To estimate the prevalence of drug interactions and associated factors among older adults followed up in a Comprehensive Medication Management Service at Primary Care.

**Methods:**

Firstly, the Beers criteria 2015 was used to define drug interactions; later, drug interactions proposed by Dumbreck for patients with diabetes, depression, and heart failure were evaluated. The associated factors were assessed by univariate (Pearson's *χ*^2^) and multivariate analyses (logistic regression). The significance level of 5% was set for all analyses.

**Results:**

The mean age of the studied population was 70.2±7.8 years; 52.2% were between 60 and 69 years, and 61.3% were female. Among the older adults, 94.5% used two or more drugs (condition for the occurrence of drug-drug interaction). The prevalence of drug interaction according to the Beers criteria was 4.9%. After multivariate analysis, diseases of the central nervous system, arrhythmia, number of medications, and female sex were positively associated with drug interaction. The prevalence of drug interaction according to Dumbreck was 27.2%. After multivariate analysis, the number of medications, the presence of heart failure, and Charlson comorbidity index greater than 1 were conditions positively associated with drug interactions.

**Conclusion:**

The holistic and individualized approach used in comprehensive medication management services for older patients is important, considering the prevalence of drug interactions and the need to minimize adverse events.

## INTRODUCTION

Demographic transition has been observed in several countries, which are at different stages and present processes with unique features. In Brazil, the increase in life expectancy and decrease in fertility rates – which are determinants in this transition process, occurred only as early as 1960.^(^^1^^)^ Currently, there is a fast demographic aging at national level. Some projections estimate that, by 2030, the aged population will be approximately 41.5 million.^(^^2^^)^

With increased life expectancy and the growing number of older people, the profile of diseases changes, with greater prevalence of chronic and degenerative diseases that require continuous use of medications.^(^^3^^,^^4^^)^ The use of multiple medications is common among older patients, putting these individuals at risk of using potentially inappropriate medications, as well as of drug interactions.^(^^3^^,^^5^^)^

Furthermore, aging brings physiological changes that influence the pharmacokinetics and pharmacodynamics of most drugs often used by older adults. These changes increase the frequency of adverse events associated with the use of drugs and the interactions among them.^(^^6^^)^

Potentially serious drug interactions, which are considered avoidable adverse events, may lead to severe and even lethal outcomes.^(^^7^^)^ Pedrós et al.^(^^8^^)^ estimated that one out of 30 admissions of older patients at hospital emergency departments was associated with drug-related adverse events, and about half of these was likely to be caused by drug interactions. In a meta-analysis, Dechanont et al.^(^^9^^)^ identified that 1.1% of hospital admissions were related to drug interactions. On the other hand, McDonnell et al.^(^^10^^)^ evaluated preventable adverse events in hospital admissions and found that approximately 26% of all admissions involved drug interactions, which could have been prematurely recognized by pharmacists and primary care health workers.

## OBJECTIVE

To estimate the prevalence of drug interactions and associated factors among older individuals followed up in a Comprehensive Medication Management Service at Primary Care.

## METHODS

This is a cross-sectional study carried out to estimate the prevalence of drug interactions, identify the most frequent interactions, and detect the associated factors.

All patients aged ≥60 years, as defined by the World Health Organization (WHO) for older people in developing countries, registered at the Comprehensive Medication Management Service (CMM) at the primary care setting, in the city of Lagoa Santa (State of Minas Gerais), between April 2015 and February 2016, who were taking at least two medications during the follow-up period, were included (n=436). Lagoa Santa is a city in the State of Minas Gerais (Brazil), located in the metropolitan region of Belo Horizonte, 35km away from the capital city.

The following data were collected: sex, age, health problems, and medications used (prescribed and non-prescribed) classified according to the Anatomical Therapeutic Chemical Code Classification System (ATC). The Charlson comorbidity index (CCI) was also calculated for each of the patients included in the CMM service.^(^^11^^)^ All data were collected retrospectively from the records of the CMM Service at the primary care setting, which are documented by the pharmacist responsible for taking caring of patients.

The data of the used drugs (prescribed and non-prescribed) were identified in the first or second visits, with the pharmacist checking the prescription and drug package, considering that often the complete list of medications used by the patient at the beginning of the care process was determined by the pharmacist only at the second visit. Among these drugs, potentially inappropriate drug interactions for the older adults were identified. For this purpose, the table of potentially clinically important non-anti-infective drug-drug interactions that should be avoided in older adults, according to the 2015 Beers criteria update, was used.^(^^12^^)^

Moreover, patients with diagnosis of heart failure, depression and type-2 *diabetes mellitus* (DM) were also identified. In the drug therapy of these patients, the potentially severe drug-drug interactions proposed by Dumbreck et al.^(^^13^^)^ were identified. This study identified the relevant interactions involving drugs used in the clinical guidelines of the National Institute of Health and Care Excellence (NICE) for the three diseases, in addition to the medications listed in nine other protocols of common diseases that patients present as comorbidities: atrial fibrillation, osteoarthritis, chronic obstructive pulmonary disease, hypertension, secondary prevention after myocardial infarction, dementia, rheumatoid arthritis, chronic kidney disease, and neuropathic pain.

The descriptive analysis of data was conducted by determining the absolute and relative frequencies of the qualitative variables, and the mean and standard deviation of the quantitative variables.

Two dependent variables were defined for the univariate and multivariate analyses: the identification of at least one drug interaction according to the Beers criteria;^(^^12^^)^ and the identification of at least one drug interaction according to Dumbreck et al.^(^^13^^)^

The following independent variables were evaluated by taking both dependent variables: sex, age (60-69 years *versus* 70-98 years), number of health problems (0-4 health problems *versus* 5 or more), number of medications used (2-5 medications *versus* 6 or more), CCI (0 *versus* 1 or more), diagnosis of hypertension (yes *versus* no), diagnosis of DM (yes *versus* no), diagnosis of heart failure (yes *versus* no), diagnosis of depression disorder (yes *versus* no), diagnosis of arrhythmia (yes *versus* no), and central nervous system diseases (yes *versus* no). The quantitative variables (age, number of health problems selected, number of medications and CCI) were divided according to their median. For the variable CCI, we chose to use the original version of the score without including the age group in the calculation of severity, since age is also used as an independent variable in multivariate models.

For univariate analysis, Pearson´s *χ*^2^ test was used, or Fisher's exact test, when the expected value of one or more cells was five or less. Independent variables with p<0.20 in the univariate analysis were included in the multivariate model calculated by stepwise logistic regression. The Hosmer-Lemeshow test was used to evaluate the quality of adjustment of the multivariate model. The univariate and multivariate analyses were based on the odds ratio (OR) and the respective 95% confidence interval (95% CI), as estimated by logistic regression. The level of significance of 5% was the criterion adopted to identify the characteristics independently associated to the dependent variable.

The information collected was entered into the Excel^®^ spreadsheets, and then transferred to and organized in a Stata^®^ 12 database software (Stata Corp. College Station, USA).

This study is part of the Clinical, Economic, Humanistic, Cultural, and Educational Aspects of Comprehensive Medication Management Services, at the Brazilian National Health System project, approved by the Internal Review Board of the *Universidade Federal de Minas Gerais*, under protocol CAAE: 25780314.4.0000.5149.

## RESULTS

Older patients who used two or more medications (n=408) - condition necessary for drug interaction, were included in the study. The mean age was 70.2±7.8 years (minimum: 60, maximum: 98 years), and most were in the age range 60-69 years (52.2%, n=213), and were female (61.3%, n=250). The mean number of health problems was 3.4±1.5, and, regarding drug use, the majority used five or more medications (54.9%; n=184) with a mean of 5.1±2.3 medications.

Considering the drug interactions according to the Beers criteria, 22 interactions were identified in 20 patients, with a prevalence of 4.9% in the studied population. The association of three or more drugs acting in the central nervous system was the most common interaction, found in 3.2% of the older adults (Table 1).

**Table 1:** Potentially clinically important non-anti-infective drug-drug interactions that should be avoided in older adults, according to Beers criteria

Drug interaction	Potential adverse event	Interactions n (%)
Use of three or more drugs that act on the CNS	Falls and fractures	13 (59.1)
Association with anti-cholinergic drugs	Cognitive decline	4 (18.2)
Alpha-1 antagonist *versus* loop diuretics	Urinary incontinence in older women	4 (18.2)
Warfarin *versus* amiodarone	Bleeding	1 (4.5)
	Total	22 (100)

CNS: central nervous system.

In the multivariate analysis of association with the presence of drug-drug interactions proposed by Beers, the existence of a central nervous system disease was the most strongly associated variable (OR=10.8; 95%CI: 3.82-30.57; p>0.05). Arrhythmia, female sex and use of six or more drugs also remained associated with the presence of the drug interactions proposed by Beers in multivariate analyses (Table 2).

**Table 2:** Univariate and multivariate analyses of the factors associated to drug-drug interactions, according to the Beers criteria

Variables		Drug-drug interactions*		Univariate		Multivariate	
		Yes n (%)	No n (%)	OR (95%CI)†	p value p‡	OR (95%CI)§	p value p§
Sex							
	Male	3 (15.0)	155 (39.9)	1		1	
	Female	17 (85.0)	233 (60.1)	3.77 (1.08-13.08)	0.026	4.45 (1.18-16.73)	0.027
Age							
	60-69	12 (5.6)	201 (94.4)	1			
	>70	8 (4.1)	187 (95.9)	0.72 (0.28-1.79)	0.474		
Number of drugs							
	2-5	6 (2.3)	253 (97.7)	1		1	
	>6	14 (9.4)	135 (90.6)	4.37 (1.64-11.64)	0.001	3.41 (1.21-9.57)	0.020
Number of health problems							
	0-4	13 (4.0)	312 (96.0)	1			
	5 or more	7 (8.4)	76 (91.6)	2.21 (0.85-5.73)	0.095		
Charlson comorbidity index							
	0	10 (5.4)	177 (94.7)	1			
	>1	10 (4.5)	211 (95.5)	0.84 (0.34-2.06)	0.701		
Hypertension							
	No	3 (7.0)	40 (93.0)	1			
	Yes	17 (4.5)	348 (95.3)	0.65 (0.18-2.32)	0.505		
Diabetes							
	No	14 (5.7)	234 (94.3)	1			
	Yes	6 (3.7)	154 (96.3)	0.65 (0.24-1.73)	0.387		
Heart failure							
	No	19 (4.9)	366 (95.1)	1			
	Yes	1 (4.3)	22 (95.7)	0.88 (0.11-6.85)	0.899		
Depression							
	No	15 (75.0)	366 (94.3)	1			
	Yes	5 (25.0)	22 (5.7)	5.54 (1.85-16.65)	0.001		
Arrhythmia							
	No	18 (90.0)	380 (97.9)	1		1	
	Yes	2 (10.0)	8 (2.1)	5.27 (1.04-26.67)	0.025	9.46 (1.57-56.72)	0.014
Central nervous system disease							
	No	11 (55.0)	355 (91.5)	1		1	
	Yes	9 (45.0)	33 (8.5)	8.80 (3.40-22.77)	0.000	10.8 (3.82-30.57)	0.000

* At least one drug interaction found in the first or second visits;

† estimated by logistic regression;

‡ estimated by Pearson's *χ*^2^ test;

§ estimated by stepwise logistic regression.

OR: odds ratio; 95%CI: 95% confidence interval.

Regarding the drug interactions proposed by Dumbreck et al.,^(^^13^^)^ 210 interactions were identified in 111 patients (27.2%), and 11.5% had 2 or more drug interactions. Considering the disease involved in the interaction, among the patients with DM, 150 interactions were identified (71.4%); with heart failure, 50 (23.8%); and with depression, 10 (4.8%). The three most common interactions were in patients with DM, mostly involving angiotensin II receptor antagonists (AT1 subtype) and diuretics used to treat hypertension, present in the drug therapy of 11.8% of patients evaluated; angiotensin converting enzyme (ACE) inhibitors and diuretics used to treat hypertension were found in 7.6% of patients evaluated; and calcium channel blockers and statins, in 6.4% (Table 3).

**Table 3:** Drug interactions as proposed by Dumbreck et al.^(^^13^^)^

Drug interaction		Potential adverse event	n (%)
*Diabetes mellitus*			
Angiotensin II receptor antagonists (AT1 subtype) *versus* diuretics to treat hypertension		Hypotensive effect	48 (22.9)
ACE inhibitors *versus* diuretics to treat hypertension		Hypotensive effect	31 (14.8)
Calcium channel blockers *versus* statin		Myopathy	26 (12.4)
Beta blockers *versus* calcium channel blockers		Bradycardia	21 (10.0)
Alpha antagonists *versus* diuretics to treat hypertension		Hypotensive effect	4 (1.9)
Beta blockers *versus* alpha antagonists		Hypotensive effect	3 (1.4)
Alpha antagonists *versus* calcium channel blockers		Hypotensive effect	3 (1.4)
Angiotensin II receptor antagonists (AT1 subtype) *versus.* spironolactone		Hyperkalemia	3 (1.4)
Fibrates *versus* statins		Myopathy	3 (1.4)
ACE inhibitors *versus* spironolactone		Hyperkalemia	2 (1.0)
Sulfonylurea *versus* NSAID		Abnormal PC*, requiring dose adjustment or cautious monitoring	2 (1,0)
Alpha antagonists *versus* spironolactone		Hypotensive effect	1 (0.5)
Tadalafil *versus* alpha antagonists		Hypotensive effect	1 (0.5)
Digoxin *versus* diuretics to treat hypertension		Hypokalemia	1 (0.5)
Sulfonylurea *versus* warfarin		Abnormal PC*, requiring dose adjustment or cautious monitoring	1 (0.5)
Depression			
	SSRI *versus* acetylsalicylic acid	Bleeding	2 (1.0)
	Venlafaxine *versus* acetylsalicylic acid	Bleeding	2 (1.0)
	SSRI *versus* tricyclic drugs	Ventricular arrhythmias	2 (1.0)
	SSRI *versus* antipsychotics	Ventricular arrhythmias	2 (1.0)
	SSRI *versus* clopidogrel	Bleeding	1 (0.5)
	Antipsychotics *versus* diuretics	Ventricular arrhythmias	1 (0.5)
Heart failure			
	ACE inhibitors *versus* to treat hypertension	Hypotensive effect	11 (5.2)
	Angiotensin II receptor antagonists (AT1 subtype) *versus* diuretics to treat hypertension	Hypotensive effect	9 (4.3)
	Digoxin *versus* diuretics to treat hypertension	Hypokalemia	6 (2.9)
	ACE inhibitors *versus* spironolactone	Hyperkalemia	5 (2.4)
	Angiotensin II receptor antagonists (AT1 subtype) *versus* spironolactone	Hyperkalemia	5 (2.4)
	Amlodipine *versus* simvastatin	Myopathy	4 (1.9)
	Digoxin *versus* spironolactone	Abnormal PC*, requiring dose adjustment or cautious monitoring	3 (1.4)
	Acetylsalicylic *versus* SSRI	Bleeding	2 (1.0)
	Beta blockers (HF) *versus* nifedipine	Bradycardia	2 (1.0)
	Beta blockers (HF) *versus* alpha antagonists	Hypotensive effect	1 (0.5)
	Amlodipine *versus* alpha antagonists	Hypotensive effect	1 (0.5)
	Beta blockers (HF) *versus* amiodarone	Bradycardia	1 (0.5)
		Total	210 (100)

ACE: angiotensin converting enzyme; NSAID: non-steroidal anti-inflammatory drugs; PC: clinically significant in plasma concentration; SSRI: selective serotonin reuptake inhibitors; HF: heart failure.

In the multivariate analyses of the factors associated with drug interactions as proposed by Dumbreck et al.,^(^^13^^)^ CCI greater than 1 was the most strongly associated variable (OR=23.52; 95%CI: 9.18-60.28; p>0.05). Heart failure and use of six or more drugs were also associated with drug interactions as proposed by Dumbreck et al.^(^^13^^)^ in the multivariate analyses (Table 4).

**Table 4:** Univariate and multivariate analyses of the factors associated to potentially severe drug-drug interactions described in the study by Dumbreck et al.^(^^13^^)^

Variables		Drug-drug interactions*		Univariate		Multivariate	
		Yes n (%)	No n (%)	OR (95%CI)†	p value‡	OR (95%CI)§	p value§
Sex							
	Male	40 (36.0)	118 (39.7)	1			
	Female	71 (64.0)	179 (60.3)	1.17 (0.74-1.83)	0.389		
Age							
	60-69	61 (55.0)	152 (51.2)	1			
	>70	50 (45.0)	145 (48.8)	0.86 (0.55-1.33)	0.416		
Number of drugs							
	2-5	40 (36.1)	219 (73.7)	1		1	
	>6	71 (63.9)	78 (26.3)	4.98 (3.12-7.94)	0.000	3.50 (2.02-6.08)	0.000
Number of health problems							
	0-4	71 (63.9)	254 (85.5)	1			
	>5	40 (36.1)	43 (14.5)	3.33 (2.01-5.51)	0.000		
Charlson comorbidity index							
	0	5 (4.5)	182 (61.3)	1		1	
	1 a 6	106 (95.5)	115 (38.7)	33.55 (13.27-84.77)	0.000	23.52 (9.18-60.28)	0.000
Hypertension							
	No	4 (3.6)	39 (13.1)	1			
	Yes	107 (96.4)	258 (86.9)	5.27 (1.04-26.67)	0.002		
Diabetes							
	No	17 (15.3)	231 (77.8)	1			
	Yes	94 (84.7)	66 (22.2)	8.80 (3.40-22.77)	0.000		
Heart failure							
	No	90 (81.1)	295 (99.3)	1		1	
	Yes	21 (18.9)	2 (0.7)	34.41 (7.91-149.61)	0.000	12.26 (2.72-55.17)	0.001
Depression							
	No	100 (90.1)	281 (94.6)	1			
	Yes	11 (9.9)	16 (5.4)	1.93 (0.87-4.30)	0.102		
Arrhythmia							
	No	110 (99.1)	288 (97.0)	1			
	Yes	1 (0.9)	9 (3.0)	0.29 (0.04-2.32)	0.232		
Central nervous system disease							
	No	99 (89.2)	267 (89.9)	1			
	Yes	12 (10.8)	30 (10.1)	1.07 (0.53-2.19)	0.128		

* Presence of at least one drug interaction identified in the first or second visits;

† estimated by logistic regression;

‡ estimated by Pearson's *χ*^2^ test;

§ estimated by stepwise logistic regression.

OR: odds ratio; 95%CI: 95% confidence interval.

## DISCUSSION

The prevalence of drug interactions identified according to the Beers criteria was low in our study (4.9%), and lower than that found by Bo et al.,^(^^14^^)^ among older adults being discharged from hospital in an Italian study (7.8%). To our knowledge, that was the only study to use the Beers criteria with the same purpose of this study.^(^^12^^,^^14^^)^ Despite the low prevalence, it should be noted that the type of interactions detected by the Beers criteria presents a considerable potential to cause severe harm to the geriatric population.^(^^12^^)^

The most frequent interaction according to the Beers criteria in our study (3.2%) was with three or more drugs that act on the central nervous system, which is associated with an increased risk of falls and fractures, especially in the older people with a history of falls.^(^^12^^,^^15^^)^ Several studies with older patients demonstrated the association of drugs that act on the central nervous system with falls and fractures.^(^^16^^,^^17^^)^ We, therefore, emphasize the need to comprehensively manage drug therapy in older patients, with a focus on reducing the use of drugs that act on the central nervous system, as well as choosing medications associated with lower risk and lower effective doses when indicated.^(^^15^^,^^16^^)^

The association of anticholinergic drugs and the interaction between alpha-1 antagonists and loop diuretics were also identified in four patients (1.0% prevalence for each interaction). The anticholinergic cognitive burden of drugs is assessed by the affinity for muscarinic receptors and their clinically relevant negative cognitive effects, being related to the number of anticholinergic drugs in use.^(^^18^^)^ Hence, the use of several drugs with this property, however of low intensity, significantly increases the risk of cognitive impairement and dementia.^(^^15^^,^^18^^)^ On the other hand, the drug interaction between alpha-1 antagonists and loop diuretics puts the older adults at higher risk of developing urinary incontinence, and consequently, more serious adverse events, such as falls and fractures.^(^^19^^)^

In the multivariate analysis, Bo et al.^(^^14^^)^ evaluated the drug interactions and the use of potentially inappropriate medications and, as in this study, identified an association between the number of drugs in use and drug interactions. Female patients were also the majority in the Italian study, however no association between drug interactions and sex was identified. On the other hand, in the present study, among the patients with drug interactions as proposed by Beers, female sex, central nervous system diseases and arrhythmias were factors positively and strongly associated with the drug interactions. This shows the importance of careful evaluation of such interactions among older patients with this profile.^(^^12^^)^ These groups of associated diseases, those involving the central nervous system and arrhythmias, have prognoses and treatment regimens that are complex and could make the management by primary care providers difficult. These conditions encompass important criteria for referral; however, further studies applying the Beers criteria for drug interactions are necessary to evaluate its applicability.^(^^12^^)^

In our search, we did not identify studies evaluating interactions between two drugs in the presence of specific diseases, or the list of drug interactions proposed by Dumbreck et al.^(^^13^^)^ We observed a high prevalence of disease-drug-drug interactions (27.2%), which was higher than the prevalence we found using the Beers criteria (4.9%) and of studies that evaluated disease-drug or drug-drug interactions with other lists of interactions, including in Brazil, ranging from 7.8 to 18.9%.^(^^12^^,^^14^^,^^17^^,^^20^^,^^21^^)^

Among diabetic patients, the two most frequent interactions were between angiotensin II receptor antagonists and ACE inhibitors with diuretics to treat hypertension, which are interactions of similar mechanisms, due to the pharmacodynamics of the classes involved. In patients with heart failure, the most frequent interaction was between ACE inhibitors and diuretics to treat hypertension. For patients with diabetes and heart failure, the risk of hypotension due to the association of ACE inhibitors or angiotensin II receptor antagonists with diuretics to treat hypertension becomes even more clinically relevant.^(^^13^^)^ This hypotension is a consequence of the sodium depletion produced by the ACE inhibitor that is potentiated by the additive action of the diuretic. Such reaction is more significant in the beginning of a combination treatment.^(^^22^^,^^23^^)^ Although this association increases natriuresis, ACE inhibitors can also reduce glomerular filtration, diuresis, and the natriuretic response to diuretics.^(^^24^^)^

Other drug interactions most commonly identified among diabetic patients were between beta-blockers and calcium channel blockers, and between statins and calcium channel blockers, with the respective adverse events, increased risk of bradycardia, and myopathy/rhabdomyolysis. The association of beta-blockers and calcium channel blockers has great therapeutic value, decreasing mortality in patients with ischemic syndromes.^(^^23^^,^^25^^)^ Nevertheless, there is a consequent blockade of reflex beta-adrenergic responses, which may increase therapeutic efficacy and also cause adverse events, such as bradycardia and hypotension. Older patients or individuals with other cardiovascular comorbidities are more susceptible and require dose adjustment in order to achieve control of symptoms.^(^^24^^,^^25^^)^ We also underscore that, among the older adults evaluated, interactions involving beta-blockers and non-dihydropyridine calcium channel blockers were detected, further increasing the risk of adverse events related to bradycardia.

Among the statin group, simvastatin has greater potential for interaction with other classes, especially with calcium channel blockers, with an increased risk of myopathy and rhabdomyolysis, which are severe events, despite their low prevalence.^(^^23^^,^^24^^)^ Nguyen et et al.^(^^26^^)^ evaluated the use of statins and risk factors for myopathy and rhabdomyolysis, and identified that diabetes and cardiovascular disease are among the co-morbidities with the higher associated risk. For patients with these comorbidities and indication of concurrent administration, adjustment of the statin dose is also recommended: for the combination with diltiazem and verapamil, the dose of simvastatin should not exceed 10mg per day; with amlodipine, not exceed 20mg per day.^(^^24^^)^

In the multivariate analyses of disease-drug-drug interactions proposed by Dumbreck et al.,^(^^13^^)^ CCI was the variable most strongly associated with the detection of interactions (OR = 23.52; 95%CI: 9.18 – 60.28; p>0.05). In the study by Teramura-Grönblad et al.,^(^^27^^)^ who evaluated only drug-drug interactions, CCI had no significant association with drug interactions. This result may be related to the presence of diabetes with complications and heart failure among the 17 comorbidities involved in the calculation of CCI.

Other variables associated with the multivariate analysis of disease-drug-drug interactions proposed by Dumbreck et al.^(^^13^^)^ were the use of six or more drugs, and the presence of heart failure. Our results coincide with those of other studies of non-hospitalized patients, which identified that the use of multiple drugs is associated with drug interactions^(^^21^^,^^27^^,^^28^^)^ and also to the occurrence of adverse events resulting from these interactions.^(^^29^^)^ Cardiovascular diseases have been associated with drug interactions,^(^^27^^,^^28^^)^ which often require the combination of drugs in the treatment, as well as in patients with heart failure. Busa et al.^(^^30^^)^ also identified a correlation between an increased number of drugs prescribed and an increase in drug interactions. In addition, drugs that act on the cardiovascular system was the main group involved in the drug interactions identified.

Regardless of the low prevalence of interactions identified for both references used, it is important to note that pharmacokinetic and pharmacodynamic changes associated with aging and illnesses related to the health problems, adopted as parameters for Dumbreck et al.^(^^13^^)^ are common. Reduced metabolism and excretion of various drugs and metabolites, thinning of the blood-brain barrier and greater responsiveness to central nervous system depressants are some examples. These changes potentiate the occurrence of adverse events resulting from drug interactions, intensifying the need for meticulous evaluation of drug therapy among geriatric patients.^(^^6^^,^^13^^)^ On the other hand, it is important to note that often the prescription of two or more drugs that interact with each other is necessary to meet the specific demands of the patient, and the prescriber should decide whether to include them in the patient's therapy. The multiprofessional team should monitor.

According to our search, this study is among the first to point out the prevalence of potentially clinically important non-anti-infective drug-drug interactions that should be avoided in the older adults. It is also the first to evaluate the prevalence of disease-drug-drug interactions as proposed by Dumbreck et al.^(^^13^^)^

This study has the limitation of retrospectively applying the interactions of the 2015 version of the Beers criteria,^(^^12^^)^ as well as the interactions proposed by Dumbreck et al.^(^^13^^)^ Another limitation was the non-assessment of frailty of older adults included in it, since this factor has considerable relevance regarding the potential clinical consequences of drug interactions.

## CONCLUSION

Among the older patients evaluated, drug-drug interactions were found according to the Beers criteria, with significant association with the use of multiple medications, female sex and central nervous system disease. Regarding the disease-drug-drug interactions as proposed by Dumbreck et al. these interactions were significantly associated with the use of multiple medications, heart failure, and the Charlson comorbidity index greater than 1.

It is worth emphasizing the importance of a holistic and individualized approach to the management of drug therapy in older patients, as provided in Comprehensive Medication Management services, which assess the totality of the individual as well as the drugs in use, drug interactions and multiple health problems, with a focus on prolonging longevity, minimizing unnecessary drug use, and decreasing adverse events and costs.
