# Supplementary material for: Drug interactions among older adults followed up in a comprehensive medication management service at Primary Care
Source: Einstein (Sao Paulo). 2019 Aug 19;17(4):eAO4725. doi: 10.31744/einstein_journal/2019AO4725 (PMC6706225; doi:10.31744/einstein_journal/2019AO4725)
Supplement: Translation

# Translation: Interações medicamentosas entre idosos acompanhados em serviço de gerenciamento da terapia medicamentosa da Atenção Primária

**Authors:** Tayane Oliveira dos Santos, Mariana Martins Gonzaga do Nascimento, Yone Almeida Nascimento, Grazielli Cristina Batista de Oliveira, Ursula Carolina de Morais Martins, Danielle Fernandes da Silva, Djenane Ramalho de Oliveira

**Affiliations:** 1 Centro de Estudos em Atenção Farmacêutica, Faculdade de Farmácia, Universidade Federal de Minas Gerais, Belo Horizonte, MG, Brasil

**Keywords:** Interações medicamentosas, Idoso, Conduta do tratamento medicamentoso, Lista de medicamentos potencialmente inapropriados, Farmacêuticos

## Abstract

**Objetivo:**

Estimar a prevalência de interações medicamentosas entre idosos acompanhados em um Serviço de Gerenciamento da Terapia Medicamentosa na Atenção Primária e fatores associados.

**Métodos:**

Para definir as interações medicamentosas, foi utilizado o critério de Beers de 2015 e, em um segundo momento, as interações medicamentosas propostas por Dumbreck para pacientes com diabetes, depressão e insuficiência cardíaca. A análise dos fatores associados foi realizada por análise univariada (*χ*^2^ de Pearson) e multivariada (regressão logística). Foi adotado o nível de significância de 5% para todas as análises.

**Resultados:**

A média da idade da população foi de 70,2±7,8 anos, 52,2% estavam na faixa etária de 60 a 69 anos, e 61,3% eram do sexo feminino. Dentre os idosos, 94,5% usavam dois medicamentos ou mais (condição para que ocorressem interações medicamentosas). A prevalência de interações medicamentosas dos critérios de Beers foi de 4,9%. Após análise multivariada, doença do sistema nervoso central, arritmia, número de medicamentos e sexo feminino foram positivamente associados com presença de interações medicamentosas. A prevalência das interações medicamentosas propostas por Dumbreck foi de 27,2%. Após análise multivariada, número de medicamentos, insuficiência cardíaca e índice de comorbidade de Charlson superior a 1 foram positivamente associados com presença de interações medicamentosas.

**Conclusão:**

A abordagem holística e individualizada no gerenciamento da terapia medicamentosa de pacientes idosos é importante, tendo em vista a prevalência das interações medicamentosas e a necessidade de minimizar consequentes eventos adversos.

## INTRODUÇÃO

A transição demográfica vem sendo observada em diversos países no mundo, que se encontram em estágios diferentes e apresentam processos com características peculiares. No Brasil, o aumento da expectativa de vida e a diminuição da fecundidade, determinantes neste processo de transição, foram verificados apenas a partir de 1960,^(^^1^^)^ e, atualmente, identifica-se acelerado envelhecimento demográfico nacional. Projeções estimam que, em 2030, a população idosa figurará com cerca de 41,5 milhões de indivíduos.^(^^2^^)^

Com o incremento da expectativa de vida da população e o também crescente número de idosos, surge uma mudança do perfil de doenças, com aumento da prevalência das doenças crônico-degenerativas, que envolvem uso contínuo de medicamentos.^(^^3^^,^^4^^)^ É comum identificar, entre a população idosa, o uso de múltiplos medicamentos, o que expõe o paciente aos riscos decorrentes do uso de medicamentos potencialmente inadequados e das interações medicamentosas.^(^^3^^,^^5^^)^

Ainda, o envelhecimento é acompanhado por mudanças fisiológicas que influenciam na farmacocinética e na farmacodinâmica de grande parte dos fármacos utilizados com frequência por idosos. Essas alterações aumentam a frequência de eventos adversos associados ao uso de medicamentos e às interações entre eles.^(^^6^^)^

Interações medicamentosas potencialmente graves, que são consideradas eventos adversos evitáveis, podem apresentar desfechos graves e até fatais.^(^^7^^)^ Pedrós et al.^(^^8^^)^ por exemplo, estimaram que 1 em cada 30 admissões de pacientes idosos em setores de urgência hospitalar é relacionada a eventos adversos relacionados ao uso de medicamentos, sendo que cerca de metade dessas é suspeita de ter sido ocasionada por interações. Em metanálise, Dechanont et al.^(^^9^^)^ identificaram que 1,1% das admissões hospitalares estiveram associadas com interações medicamentosas. Por outro lado, McDonnell et al.^(^^10^^)^ avaliaram eventos adversos preveníveis em admissões hospitalares e identificaram que aproximadamente 26% do total das admissões envolveu interações medicamentosas que poderiam ter sido reconhecidas precocemente pelos farmacêuticos e por profissionais de saúde da Atenção Primária.

## OBJETIVO

Estimar a prevalência de interações medicamentosas entre idosos acompanhados em um Serviço de Gerenciamento da Terapia Medicamentosa na Atenção Primária e seus fatores associados

## MÉTODOS

Estudo transversal para estimativa de prevalência de interações medicamentosas, identificação das interações mais frequentes e detecção de fatores associados a elas.

Foram incluídos, no presente estudo, todos os pacientes com 60 anos ou mais, conforme definição da Organização Mundial da Saúde (OMS) para idosos em países em desenvolvimento, inseridos no Serviço de Gerenciamento da Terapia Medicamentosa da Atenção Primária à saúde do município de Lagoa Santa (MG), no período entre abril de 2015 e fevereiro de 2016, que utilizavam pelo menos dois medicamentos durante o período de acompanhamento (n=436). Lagoa Santa é um município do Estado de Minas Gerais (Brasil), localizado na região metropolitana de Belo Horizonte, a 35km da capital.

Os seguintes dados foram coletados: sexo, idade, problemas de saúde e medicamentos utilizados (prescritos e não prescritos) classificados de acordo com o sistema *Anatomical Therapeutic Chemical Code Classification System* (ATC). Também foi calculado o índice de comorbidade de Charlson para cada um dos pacientes incluídos no serviço.^(^^11^^)^ Todos os dados foram coletados retrospectivamente nos prontuários referentes aos atendimentos do Serviço de Gerenciamento da Terapia Medicamentosa, que é preenchido pelo farmacêutico responsável pelo acompanhamento do paciente.

Os dados dos medicamentos utilizados (prescritos e não prescritos) foram referentes àqueles identificados na primeira ou segunda consulta, com conferência da prescrição e da embalagem do medicamento pelo farmacêutico, considerando que frequentemente a lista completa de medicamentos em uso pelo paciente no início do acompanhamento era acessada pelo farmacêutico apenas na segunda consulta. Dentre estes medicamentos, foram identificadas as interações medicamentosas potencialmente inadequadas para idosos presentes nas farmacoterapias dos pacientes. Para tal, empregou-se a tabela de interações medicamento-medicamento não anti-infecciosos potencialmente clinicamente importantes que devem ser evitadas em idosos atualizada em 2015 segundo o critério de Beers.^(^^12^^)^

Adicionalmente, pacientes com diagnósticos de insuficiência cardíaca, depressão e *diabetes mellitus* (DM) tipo 2 também foram identificados. Na farmacoterapia desses pacientes, foram identificadas as interações medicamentosas potenciais graves do tipo medicamento-medicamento propostas por Dumbreck et al.^(^^13^^)^ Este estudo identificou as interações relevantes envolvendo os medicamentos empregados nas diretrizes clínicas do *National Institute of Health and Care Excellence* (NICE) para as três doenças, além dos medicamentos constantes em outros nove protocolos de condições mais comuns que os pacientes apresentam como comorbidade: fibrilação atrial, osteoartrite, doença pulmonar obstrutiva crônica, hipertensão arterial sistêmica, prevenção secundária após infarto do miocárdio, demência, artrite reumatoide, doença renal crônica e dor neuropática.

A análise descritiva dos dados foi realizada mediante determinação das frequências absolutas e relativas das variáveis qualitativas, e média e desvio padrão das variáveis quantitativas.

Para as análises univariada e multivariada, definiram-se duas variáveis dependentes: a identificação de pelo menos uma interação medicamentosa de acordo com o critério de Beers;^(^^12^^)^ e a identificação de pelo menos uma interação medicamentosa de acordo com Dumbreck et al.^(^^13^^)^

As seguintes variáveis independentes foram avaliadas tomando ambas as variáveis dependentes: sexo, idade (60-69 anos *versus* 70-98 anos), número de problemas de saúde (0-4 problemas de saúde *versus* 5 ou mais), número de medicamentos utilizados (2-5 medicamentos *versus* 6 ou mais), índice de comorbidade de Charlson (0 *versus* 1 ou mais), diagnóstico de hipertensão (sim *versus não*), diagnóstico de DM (sim *versus não*), diagnóstico de insuficiência cardíaca (sim *versus* não), diagnóstico de transtorno depressivo (sim *versus* não), diagnóstico de arritmia (sim *versus* não), e doenças do sistema nervoso central (sim *versus* não). As variáveis quantitativas (idade, número de problemas de saúde selecionados, número de medicamentos e índice de comorbidade de Charlson) foram dicotomizadas de acordo com sua mediana. Para a variável índice de comorbidade de Charlson, optou-se por utilizar a versão original do escore sem inclusão da faixa etária no cálculo de gravidade, devido ao fato da idade também ser utilizada como variável independente nos modelos multivariados.

As análises univariadas foram realizadas utilizando-se o teste do *χ*^2^ de Pearson ou o teste exato de Fisher, quando o valor esperado de uma ou mais células fosse cinco ou menos. Variáveis independentes com p<0,20 nas análises univariadas foram incluídas no modelo multivariado calculado por regressão logística *stepwise*. Foi utilizado o teste Hosmer-Lemeshow para avaliar a qualidade de ajuste do modelo multivariado. As análises univariada e multivariada foram baseadas na *odds ratio* (OR) e no respectivo intervalo de confiança de 95% (IC95%), estimados por regressão logística. O nível de significância de 5% foi o critério adotado para identificar as características independentemente associadas à variável dependente.

As informações coletadas foram digitadas no programa Excel^®^ e posteriormente transferidas e organizadas em banco de dados do *software* Stata^®^ versão 12 (*Stata Corp. College Station,* Estados Unidos).

Este estudo é parte integrante do projeto Resultados Clínicos, Econômicos, Aspectos Humanísticos, Culturais e Educacionais de Serviços de Gerenciamento da Terapia Medicamentosa no Sistema Único de Saúde, aprovado pelo Comitê de Ética em Pesquisa da Universidade Federal de Minas Gerais, sob registro CAAE: 25780314.4.0000.5149.

## RESULTADOS

Foram selecionados para o presente estudo os idosos que usavam dois medicamentos ou mais, condição para que ocorresse interação medicamentosa (n=408). Os pacientes apresentaram, em média, 70,2±7,8 anos (mínimo: 60; máximo: 98), e a maioria encontrava-se na faixa etária de 60 a 69 anos (52,2%; n=213) e era do sexo feminino (61,3%; n=250). Eles apresentavam, em média, 3,4±1,5 problemas de saúde, e, tendo em vista o uso de medicamentos, a maioria dos idosos usava cinco medicamentos ou mais (54,9%; n=184) e utilizava uma média de 5,1±2,3 medicamentos.

Considerando as interações medicamentosas de acordo com o critério de Beers, foram identificadas 22 interações em 20 pacientes, configurando prevalência de 4,9% entre os idosos da amostra. A associação de três ou mais medicamentos que atuavam no sistema nervoso central foi a interação mais comum, observada em 3,2% dos idosos (Tabela 1).

**Tabela 1:** Interações medicamento-medicamento importantes que devem ser evitadas em idosos de acordo com o critério de Beers

Interação medicamentosa	Evento adverso potencial	Interações n (%)
Uso de três ou mais medicamentos que atuam no SNC	Quedas e fraturas	13 (59,1)
Associação de anticolinérgicos	Declínio cognitivo	4 (18,2)
Antagonista alfa-1 *versus* diurético de alça	Incontinência urinária em mulheres idosas	4 (18,2)
Varfarina *versus* amiodarona	Sangramento	1 (4,5)
	Total	22 (100)

SNC: sistema nervoso central.

Na análise multivariada de associação com presença das interações medicamento-medicamento propostas por Beers, a presença de doença do sistema nervoso central foi a variável mais fortemente associada (OR=10,8; IC95%: 3,82-30,57; p>0,05). Presença de arritmia, sexo feminino e uso de seis medicamentos ou mais também se mantiveram associados com a presença das interações medicamentosas propostas por Beers nas análises multivariadas (Tabela 2).

**Tabela 2:** Análise univariadas e multivariadas dos fatores associados à presença de interações medicamento-medicamento importantes que devem ser evitadas em idosos de acordo com o critério de Beers

Variáveis		Interações medicamento-medicamento*		Univariadas		Multivariada	
		Sim n (%)	Não n (%)	RC (IC95%)†	Valor de p‡	RC (IC95%)§	Valor de p§
Sexo							
	Masculino	3 (15,0)	155 (39,9)	1		1	
	Feminino	17 (85,0)	233 (60,1)	3,77 (1,08-13,08)	0,026	4,45 (1,18-16,73)	0,027
Idade							
	60-69	12 (5,6)	201 (94,4)	1			
	>70	8 (4,1)	187 (95,9)	0,72 (0,28-1,79)	0,474		
Número de medicamentos							
	2-5	6 (2,3)	253 (97,7)	1		1	
	>6	14 (9,4)	135 (90,6)	4,37 (1,64-11,64)	0,001	3,41 (1,21-9,57)	0,020
Número de problemas de saúde							
	0-4	13 (4,0)	312 (96,0)	1			
	5 ou mais	7 (8,4)	76 (91,6)	2,21 (0,85-5,73)	0,095		
Índice de comorbidade de Charlson							
	0	10 (5,4)	177 (94,7)	1			
	>1	10 (4,5)	211 (95,5)	0,84 (0,34-2,06)	0,701		
Hipertensão							
	Não	3 (7,0)	40 (93,0)	1			
	Sim	17 (4,5)	348 (95,3)	0,65 (0,18-2,32)	0,505		
Diabetes							
	Não	14 (5,7)	234 (94,3)	1			
	Sim	6 (3,7)	154 (96,3)	0,65 (0,24-1,73)	0,387		
Insuficiência cardíaca							
	Não	19 (4,9)	366 (95,1)	1			
	Sim	1 (4,3)	22 (95,7)	0,88 (0,11-6,85)	0,899		
Depressão							
	Não	15 (75,0)	366 (94,3)	1			
	Sim	5 (25,0)	22 (5,7)	5,54 (1,85-16,65)	0,001		
Arritmia							
	Não	18 (90,0)	380 (97,9)	1		1	
	Sim	2 (10,0)	8 (2,1)	5,27 (1.04-26,67)	0,025	9,46 (1,57-56,72)	0,014
Doença do sistema nervoso central							
	Não	11 (55,0)	355 (91,5)	1		1	
	Sim	9 (45,0)	33 (8,5)	8,80 (3,40-22,77)	0,000	10,8 (3,82-30,57)	0,000

* Presença de pelo menos uma interação medicamento-medicamento identificada na primeira e segunda consultas;

† estimado por regressão logística;

‡ estimado por *χ*^2^ de Pearson;

§ estimado por regressão logística *stepwise.*

OR: *odds ratio*; IC95%: intervalo de confiança 95%.

Quanto às interações medicamentosas propostas por Dumbreck et al.,^(^^13^^)^ foram identificadas 210 interações distribuídas em 111 pacientes (27,2%), sendo que 11,5% apresentaram 2 ou mais interações medicamentosas. Considerando a doença envolvida na interação, entre os pacientes com DM, foram identificadas 150 interações (71,4%); com insuficiência cardíaca, 50 (23,8%); e com depressão, 10 (4,8%). As três interações mais comuns foram em pacientes com DM, com destaque para antagonistas de receptores de angiotensina II do subtipo AT1 e diuréticos que atuavam na pressão sanguínea, presente na farmacoterapia de 11,8% dos pacientes avaliados; inibidores da enzima conversora de angiotensina (IECA) e diuréticos que atuavam na pressão sanguínea foram encontrados em 7,6% dos pacientes avaliados; e bloqueadores dos canais de cálcio e estatinas, em 6,4% dos pacientes (Tabela 3).

**Tabela 3:** Interações medicamentosas propostas por Dumbreck et al.^(^^13^^)^

Interação medicamentosa		Evento adverso potencial	n (%)
*Diabetes mellitus*			
Antagonistas de receptores angiotensina II do subtipo AT1 *versus* diuréticos que atuam na pressão sanguínea		Efeito hipotensivo	48 (22,9)
IECA *versus* diuréticos que atuam na pressão sanguínea		Efeito hipotensivo	31 (14,8)
Bloqueadores dos canais de cálcio *versus* estatina		Miopatia	26 (12,4)
Betabloqueadores *versus* bloqueadores dos canais de cálcio		Bradicardia	21 (10,0)
Antagonistas alfa *versus* diuréticos que atuavam na pressão sanguínea		Efeito hipotensivo	4 (1,9)
Betabloqueadores *versus* antagonistas alfa		Efeito hipotensivo	3 (1,4)
Antagonistas alfa *versus* bloqueadores dos canais de cálcio		Efeito hipotensivo	3 (1,4)
Antagonistas de receptores angiotensina II do subtipo AT1 *versus* espironolactona		Hipercalemia	3 (1,4)
Fibratos *versus* estatinas		Miopatia	3 (1,4)
IECA *versus* espironolactona		Hipercalemia	2 (1,0)
Sulfonilureia *versus* AINE		Alteração CP*, requerendo ajuste de dose ou monitorização cautelosa	2 (1,0)
Antagonistas alfa *versus* espironolactona		Efeito hipotensivo	1 (0,5)
Tadalafila *versus* antagonistas alfa		Efeito hipotensivo	1 (0,5)
Digoxina *versus* diuréticos que atuam na pressão sanguínea		Hipocalemia	1 (0,5)
Sulfonilureia *versus* varfarina		Alteração CP*, requerendo ajuste de dose ou monitorização cautelosa	1 (0,5)
Depressão			
	ISRS *versus* ácido acetilsalicílico	Sangramento	2 (1,0)
	Venlafaxina *versus* ácido acetilsalicílico	Sangramento	2 (1,0)
	ISRS *versus* tricíclicos	Arritmias ventriculares	2 (1,0)
	ISRS *versus* antipsicóticos	Arritmias ventriculares	2 (1,0)
	ISRS *versus* clopidogrel	Sangramento	1 (0,5)
	Antipsicóticos *versus* diuréticos	Arritmias ventriculares	1 (0,5)
Insuficiência cardíaca			
	IECA *versus* diuréticos que atuam na pressão sanguínea	Efeito hipotensivo	11 (5,2)
	Antagonista de receptores angiotensina II do subtipo AT1 *versus* diuréticos que atuam na pressão sanguínea	Efeito hipotensivo	9 (4,3)
	Digoxina *versus* diuréticos que atuam na pressão sanguínea	Hipocalemia	6 (2,9)
	IECA *versus* espironolactona	Hipercalemia	5 (2,4)
	Antagonistas de receptores angiotensina II do subtipo AT1 *versus* espironolactona	Hipercalemia	5 (2,4)
	Anlodipino *versus* sinvastatina	Miopatia	4 (1,9)
	Digoxina *versus* espironolactona	Alteração CP*, requerendo ajuste de dose ou monitorização cautelosa	3 (1,4)
	Ácido acetilsalicílico *versus* ISRS	Sangramento	2 (1,0)
	Betabloqueadores (IC) *versus* nifedipino	Bradicardia	2 (1,0)
	Betabloqueadores (IC) *versus* Antagonistas alfa	Efeito hipotensivo	1 (0,5)
	Anlodipino *versus* antagonistas alfa	Efeito hipotensivo	1 (0,5)
	Betabloqueadores (IC) *versus* amiodarona	Bradicardia	1 (0,5)
		Total	210 (100)

IECA: inibidores da enzima conversora de angiotensina; AINE: antinflamatórios não esteroidais; CP: clinicamente significativa na concentração plasmática; ISRS: inibidores seletivos da receptação de serotonina; IC: insuficiência cardíaca.

Na análise multivariada de associação com presença das interações medicamentosas propostas por Dumbreck et al.^(^^13^^)^ o índice de comorbidade de Charlson superior a 1 foi a variável mais fortemente associada (OR=23,52; IC95%: 9,18-60,28; p>0,05). Presença de insuficiência cardíaca e uso de seis medicamentos ou mais também se mantiveram associados com a presença das interações medicamentosas propostas por Dumbreck et al.^(^^13^^)^ nas análises multivariadas (Tabela 4).

**Tabela 4:** Análises univariadas e multivariada dos fatores associados à presença de interações medicamentosas potenciais graves do tipo medicamento-medicamento descritas no estudo de Dumbreck et al.^(^^13^^)^

Variáveis		Interações medicamento-medicamento*		Univariadas		Multivariada	
		Sim n (%)	Não n (%)	RC (IC95%)†	Valor de p‡	RC (IC95%)§	Valor de p§
Sexo							
	Masculino	40 (36,0)	118 (39,7)	1			
	Feminino	71 (64,0)	179 (60,3)	1,17 (0,74-1,83)	0,389		
Idade							
	60-69	61 (55,0)	152 (51,2)	1			
	>70	50 (45,0)	145 (48,8)	0,86 (0,55-1,33)	0,416		
Número de medicamentos							
	2-5	40 (36,1)	219 (73,7)	1		1	
	>6	71 (63,9)	78 (26,3)	4,98 (3,12-7,94)	0,000	3,50 (2,02-6,08)	0,000
Número de problemas de saúde							
	0-4	71 (63,9)	254 (85,5)	1			
	>5	40 (36,1)	43 (14,5)	3,33 (2,01-5,51)	0,000		
Índice de comorbidade de Charlson							
	0	5 (4,5)	182 (61,3)	1		1	
	1 a 6	106 (95,5)	115 (38,7)	33,55 (13,27-84,77)	0,000	23,52 (9,18-60,28)	0,000
Hipertensão							
	Não	4 (3,6)	39 (13,1)	1			
	Sim	107 (96,4)	258 (86,9)	5,27 (1.04-26,67)	0,002		
Diabetes							
	Não	17 (15,3)	231 (77,8)	1			
	Sim	94 (84,7)	66 (22,2)	8,80 (3,40-22,77)	0,000		
Insuficiência cardíaca							
	Não	90 (81,1)	295 (99,3)	1		1	
	Sim	21 (18,9)	2 (0,7)	34,41 (7,91-149,61)	0,000	12,26 (2,72-55,17)	0,001
Depressão							
	Não	100 (90,1)	281 (94,6)	1			
	Sim	11 (9,9)	16 (5,4)	1,93 (0,87-4,30)	0,102		
Arritmia							
	Não	110 (99,1)	288 (97,0)	1			
	Sim	1 (0,9)	9 (3,0)	0,29 (0,04-2,32)	0,232		
Doença sistema nervoso central							
	Não	99 (89,2)	267 (89,9)	1			
	Sim	12 (10,8)	30 (10,1)	1,07 (0,53-2,19)	0,128		

* Presença de pelo menos uma interação medicamento-medicamento identificada na primeira e segunda consultas;

† estimado por regressão logística;

‡ estimado por *χ*^2^ de Pearson;

§ estimado por regressão logística *stepwise*.

OR: *odds ratio*; IC95%: intervalo de confiança 95%.

## DISCUSSÃO

A prevalência de interações medicamentosas segundo o critério de Beers identificada foi reduzida no presente estudo (4,9%) e inferior ao detectado por Bo et al.,^(^^14^^)^ entre idosos no processo de alta hospitalar em estudo italiano (7,8%), que, ao nosso conhecimento, foi o único a utilizar o critério de Beers para os mesmo objetivos deste estudo.^(^^12^^,^^14^^)^ Apesar da baixa prevalência, deve-se destacar que o tipo de interações detectado pelo critério de Beers apresenta potencial considerável de dano grave à população geriátrica.^(^^12^^)^

A interação mais frequente no presente estudo (3,2%) foi entre três medicamentos ou mais que atuam no sistema nervoso central, de acordo com o critério de Beers, e está associada com aumento do risco de queda e fraturas, principalmente em idosos com histórico de quedas.^(^^12^^,^^15^^)^ Inúmeros estudos realizados com idosos têm demostrado a associação entre medicamentos que atuam no sistema nervoso central com quedas e fraturas.^(^^16^^,^^17^^)^ Desse modo, ressalta-se a necessidade do gerenciamento da terapia medicamentosa em pacientes idosos, com enfoque na diminuição do uso de medicamentos que atuam no sistema nervoso central, bem como escolha de medicamentos associados a um menor risco e a menores doses efetivas quando indicado.^(^^15^^,^^16^^)^

A associação de medicamentos com ação anticolinérgica e a interação entre antagonistas alfa-1 e diuréticos de alça também foram identificadas em quatro pacientes (1,0% de prevalência para cada interação). A carga anticolinérgica cognitiva dos medicamentos é avaliada pela afinidade pelos receptores muscarínicos e seus efeitos cognitivos negativos clinicamente relevantes, estando relacionada ao número de medicamentos anticolinérgicos em uso.^(^^18^^)^ Assim, a presença de vários medicamentos com esta propriedade, ainda que de baixa intensidade, aumenta significativamente o risco de declínio cognitivo e demência.^(^^15^^,^^18^^)^ Já a interação medicamentosa entre antagonistas alfa-1 e diuréticos de alça expõe idosos a maior risco de desenvolvimento de incontinência urinária, e, consequentemente, a eventos adversos mais graves, como quedas e fraturas.^(^^19^^)^

Nas análises multivariadas, Bo et al.^(^^14^^)^ avaliaram as interações medicamentosas justamente de medicamentos potencialmente inapropriados e, assim como no presente estudo, identificaram associação entre número de medicamentos em uso com presença de interações medicamentosas. Pacientes do sexo feminino também eram maioria no estudo italiano, mas não foi identificada associação entre detecção de interações medicamentosas e sexo. No presente estudo, entre os pacientes com interações medicamentosas propostas por Beers, sexo feminino, presença de doenças do sistema nervoso central e presença de arritmias foram fatores positivamente associados à presença de interações medicamentosas, ressaltando a importância da avaliação criteriosa dessas interações entre pacientes idosos com este perfil.^(^^12^^)^ Esses grupos de doenças associadas, sistema nervoso central e arritmias, possuem prognósticos e esquemas de tratamentos cujas complexidades podem dificultar o manejo por profissionais da Atenção Primária, sendo importantes critérios de encaminhamento, mas ainda são necessários novos estudos aplicando as interações medicamentosas do critério de Beers para empregabilidade.^(^^12^^)^

Em nossas buscas, não foram identificados estudos que avaliaram interações entre dois medicamentos na presença de doenças específicas ou a lista de interações medicamentosas propostas por Dumbreck et al.^(^^13^^)^ Identificamos elevada prevalência das interações doença-medicamento-medicamento (27,2%), que foi superior à prevalência que identificamos nas interações do critério de Beers (4,9%) e de estudos que avaliaram interações doença-medicamento ou medicamento-medicamento com outras listas de interações, inclusive no Brasil, que variaram de 7,8 a 18,9%.^(^^12^^,^^14^^,^^17^^,^^20^^,^^21^^)^

Entre os pacientes com diabetes, as duas interações mais frequentes foram entre antagonistas dos receptores de angiotensina II e IECA com os diuréticos que atuam na pressão sanguínea, que são interações de mecanismos semelhantes, devido à farmacodinâmica das classes envolvidas. Nos pacientes com insuficiência cardíaca, a interação mais frequente foi entre IECA com os diuréticos que atuam na pressão sanguínea. Para pacientes com diabetes e insuficiência cardíaca, o risco de hipotensão devido à associação de IECA ou antagonistas dos receptores da angiotensina II com diuréticos que atuam na pressão sanguínea se torna ainda mais relevante clinicamente.^(^^13^^)^ Essa hipotensão é reflexo da depleção de sódio pelo inibidor da enzima conversora de angiotensina potencializada pela ação aditiva do diurético. Tal reação é mais significativa no início do tratamento em associação.^(^^22^^,^^23^^)^ Embora essa associação aumente a natriurese, os IECA também podem reduzir a filtração glomerular, a diurese e as respostas natriuréticas aos diuréticos.^(^^24^^)^

Outras interações medicamentosas identificadas com mais frequência entre os pacientes com diabetes foram entre betabloqueadores e bloqueadores de canais de cálcio e entre estatinas e bloqueadores dos canais de cálcio, tendo como eventos adversos, respectivamente, aumento do risco de bradicardia e miopatia/rabdomiólise. A associação de betabloqueadores e bloqueadores de canais de cálcio possui grande valor terapêutico, diminuindo a mortalidade em pacientes com síndromes isquêmicas.^(^^23^^,^^25^^)^ Entretanto, ocorre o consequente bloqueio das respostas beta-adrenérgicas reflexas, que podem aumentar a eficácia terapêutica e também os eventos adversos, como bradicardia e hipotensão. Pacientes idosos ou com outras comorbidades cardiovasculares são suscetíveis e requerem adequação das doses para apresentarem controle dos sintomas.^(^^24^^,^^25^^)^ Destacamos, ainda, que, entre os idosos avaliados, foram detectadas, especificamente, interações envolvendo betabloqueadores e bloqueadores de canais de cálcio não diidropiridínicos, trazendo ainda mais riscos de eventos adversos relacionados à bradicardia.

Entre o grupo das estatinas, a sinvastatina apresenta maior potencial de interação com outras classes, principalmente com os bloqueadores dos canais de cálcio com aumento do risco de miopatia e rabdomiólise, que são eventos graves, apesar da baixa prevalência.^(^^23^^,^^24^^)^ Nguyen et al.^(^^26^^)^ avaliaram o uso de estatinas e os fatores de risco para miopatia e rabdomiólise, e identificaram que diabetes e doença cardiovascular estão entre as comorbidades de maior risco associado. Para pacientes com essas comorbidades e indicação de coadministração, é ainda mais recomendado o ajuste da dose da estatina: para a associação com diltiazem e verapamil, a dose de sinvastatina não deve ultrapassar 10mg por dia; com anlodipino, não ultrapassar 20mg por dia.^(^^24^^)^

Nas análises multivariadas das interações doença-medicamento-medicamento propostas por Dumbreck et al.,^(^^13^^)^ o índice de comorbidade de Charlson foi a variável mais fortemente associada à detecção de interações (OR=23,52; IC95%: 9,18-60,28; p>0,05). No estudo de Teramura-Grönblad et al.,^(^^27^^)^ que avaliaram apenas interações medicamento-medicamento, o índice de comorbidade de Charlson não teve associação significativa com presença de interações medicamentosas. Nosso resultado pode estar relacionado à presença de diabetes com complicações e insuficiência cardíaca entre as 17 comorbidades envolvidas no cálculo do índice de comorbidade de Charlson.

Outras variáveis associadas nas análises multivariadas das interações doença-medicamento-medicamento propostas por Dumbreck et al.^(^^13^^)^ foram o uso de seis medicamentos ou mais, e a presença de insuficiência cardíaca. Nossos resultados coincidem com os de outros estudos com pacientes não hospitalizados, que identificaram que o uso de múltiplos medicamentos está associado à presença de interações medicamentosas^(^^21^^,^^27^^,^^28^^)^ e também à ocorrência de eventos adversos decorrentes dessas interações.^(^^29^^)^ Doenças cardiovasculares têm sido associadas com presença de interações medicamentosas^(^^27^^,^^28^^)^ que frequentemente requerem associação de medicamentos no tratamento, bem como em pacientes com insuficiência cardíaca. Busa et al.^(^^30^^)^ também identificaram correlação entre aumento do número de medicamentos prescritos e aumento da interação medicamentosas. Além disso, os medicamentos que atuam no sistema cardiovascular foram os principais grupos envolvidos nas interações medicamentosas identificadas.

Independente da baixa prevalência de interações identificadas para ambas as referências utilizadas, é importante ressaltar que são usuais alterações farmacocinéticas e farmacodinâmicas associadas aos processos de envelhecimento e de adoecimento relacionados aos problemas de saúde adotados como parâmetros para Dumbreck et al.^(^^13^^)^ São exemplos: redução do metabolismo e excreção de vários fármacos e metabólitos, afinamento da barreira hematoencefálica e maior responsividade a agentes depressores do sistema nervoso central. Essas alterações potencializam a ocorrência de eventos adversos decorrentes de interações medicamentosas, intensificando a necessidade de avaliação meticulosa da farmacoterapia entre pacientes geriátricos.^(^^6^^,^^13^^)^ Por outro lado, é importante ressaltar que, muitas vezes, a prescrição de dois ou mais medicamentos que interagem entre si é necessária para atender demandas específicas do paciente, cabendo ao prescritor a decisão terapêutica de inseri-la em sua farmacoterapia e à equipe multiprofissional realizar seu devido monitoramento.

Considerando nossas buscas, o presente estudo está entre os primeiros a apontar a prevalência das interações medicamento-medicamento não anti-infecciosos potencialmente clinicamente importantes que devem ser evitadas em idosos, além de ser o primeiro a avaliar a prevalência das interações doença-medicamento-medicamento propostas por Dumbreck et al.^(^^13^^)^

O presente estudo apresenta como limitação o fato de as interações da última versão de 2015 do critério de Beers^(^^12^^)^ e das interações propostas por Dumbreck et al.^(^^13^^)^ terem sido aplicadas retrospectivamente. Outra limitação foi a não avaliação da fragilidade dos idosos incluídos no presente estudo, uma vez que esse fator possui considerável relevância no tocante às potenciais consequências clínicas de interações medicamentosas.

## CONCLUSÃO

Entre os idosos avaliados, foram encontradas interações medicamento-medicamento propostas pelo critério de Beers, com associação significativa com uso de múltiplos medicamentos, sexo feminino e doença do sistema nervoso central. Quanto às interações doença-medicamento-medicamento propostas por Dumbreck et al. os idosos apresentaram associação significativa com uso de múltiplos medicamentos, presença de insuficiência cardíaca e índice de comorbidade de Charlson superior a 1.

Vale ressaltar a importância de uma abordagem holística e totalmente individualizada no gerenciamento da terapia medicamentosa dos pacientes idosos, como aquela realizada nos serviços de gerenciamento da terapia medicamentosa, que avalia o individuo como um todo, bem como os medicamentos em uso, as interações medicamentosas e os múltiplos problemas de saúde, com enfoque em prolongar a longevidade, reduzir sintomas, minimizar o uso desnecessário de medicamentos, e diminuir eventos adversos e custos.
